# A New Leaf Essential Oil from Endemic *Gynoxys laurifolia* (Kunth) Cass. of Southern Ecuador: Chemical and Enantioselective Analyses

**DOI:** 10.3390/plants12152878

**Published:** 2023-08-06

**Authors:** Gianluca Gilardoni, Luis Rubén Lara, Nixon Cumbicus, Omar Malagón

**Affiliations:** 1 Departamento de Química, Universidad Técnica Particular de Loja (UTPL), Calle Marcelino Champagnat s/n, Loja 110107, Ecuador; ggilardoni@utpl.edu.ec (G.G.); lrlara1@utpl.edu.ec (L.R.L.); 2 Departamento de Ciencias Biológicas y Agropecuarias, Universidad Técnica Particular de Loja (UTPL), Calle Marcelino Champagnat s/n, Loja 110107, Ecuador; nlcumbicus@utpl.edu.ec

**Keywords:** *Senecio laurifolius*, gas chromatography, mass spectrometry, enantiomers, β-cyclodextrin

## Abstract

The fresh leaves of *Gynoxys laurifolia* (Kunth) Cass. (Asteraceae), collected in the province of Loja (Ecuador), were submitted to steam distillation, producing an essential oil with a yield of 0.02% by weight. This volatile fraction, described here for the first time, was submitted to qualitative (GC–MS) and quantitative (GC–FID) chemical analyses, on two orthogonal columns (non-polar and polar stationary phase). A total of 90 components, corresponding to 95.9–95.0% by weight on the non-polar and polar stationary phase, respectively, were detected and quantified with at least one column. Major constituents (≥3%) were: germacrene D (18.9–18.0%), (*E*)-β-caryophyllene (13.2–15.0%), α-pinene (11.0–10.3%), β-pinene (4.5–4.4%), β-phellandrene (4.0–3.0%), bicyclogermacrene (4.0–3.0%), and bakkenolide A (3.2–3.4%). This essential oil was dominated by sesquiterpene hydrocarbons (about 45%), followed by monoterpene hydrocarbons (about 25–30%). This research was complemented with the enantioselective analysis of some common chiral terpenes, carried out through 2,3-diethyl-6-*tert*-butyldimethylsilyl-β-cyclodextrin and 2,3-diacetyl-6-*tert*-butyldimethylsilyl-β-cyclodextrin as stationary phase chiral selectors. As a result, (1*S*,5*S*)-(−)-β-pinene, (*R*)-(−)-α-phellandrene, (*R*)-(−)-β-phellandrene, (*S*)-(−)-limonene, (*S*)-(+)-linalyl acetate, and (*S*)-(−)-germacrene D were observed as enantiomerically pure compounds, whereas α-pinene, linalool, terpinene-4-ol, and α-terpineol were present as scalemic mixtures. Finally, sabinene was practically racemic. Due to plant wildness and the relatively low distillation yield, no industrial applications can be identified, in the first instance for this essential oil. The focus of the present study is therefore academic.

## 1. Introduction

Thanks to the presence of specialized metabolites, it is well known that plants have historically been the first source of pharmaceuticals and medicinal products. Nowadays, after more than a century of deeper and deeper investigation in search of new natural products, phytochemistry has shifted its focus to tropical countries, where new botanical species are discovered every year. In this regard, Ecuador is a world leading country in biodiversity since it appears in the list of the seventeen “megadiverse” countries [1]. For this reason, and due to historical causes, most of the native and endemic botanical species of Ecuador are still poorly studied or completely unprecedented [2,3]. In this context, the authors have been studying the phytochemistry of the Ecuadorian flora for more than twenty years, with the aim of contributing to the advance of its knowledge and discovering new secondary metabolites of chemical and pharmaceutical interest [4,5,6,7]. In the last seven years, our group has been mainly focusing on the chemical, enantiomeric, and olfactory descriptions of new essential oils (EOs), defined by the European Pharmacopoeia as “odorous products, usually of complex composition, obtained from a botanically defined plant raw material by steam distillation, dry distillation, or a suitable mechanical process without heating” [8,9,10,11,12,13].

This work is part of an academic unfunded project, dealing with the systematic chemical and enantiomeric description of new EOs from the genus *Gynoxys* Cass. (Asteraceae) of Southern Ecuador. Once this systematic investigation is completed, a statistically based comparison will be carried out on the studied species, in order to determine the existence of chemotaxonomically related botanical groups. According to the WFO Plant List database, the genus *Gynoxys* has 153 species, of which 120 are accepted, 17 unplaced, and 16 are synonyms [14]. In Ecuador, 33 species are reported, of which 23 are endemics [15]. So far, according to the literature, only five species have been studied for their EOs, three of which (*G. miniphylla*, *G. rugulosa*, and *G. buxifolia*) are a part of this project [16,17,18]. The object of the present study is the EO distilled from the leaves of *Gynoxys laurifolia* (Kunth.) Cass., an endemic Ecuadorian tree, only growing in the provinces of Loja and Azuay [15]. This species is also known by the synonym *Senecio laurifolius* Kunth, and it grows in a range of 2000–3000 m above sea level [15]. In addition to the chemical composition of *G. laurifolia* EO, this research was complemented with the enantioselective analysis of some main chiral components, in order to determine their enantiomeric excesses and, according to literature, their stereoselective biological properties.

To the best of the authors’ knowledge, this is the first chemical and enantioselective investigation of an EO from *G. laurifolia*.

## 2. Results

### 2.1. Chemical Analysis

**Figure 1:**
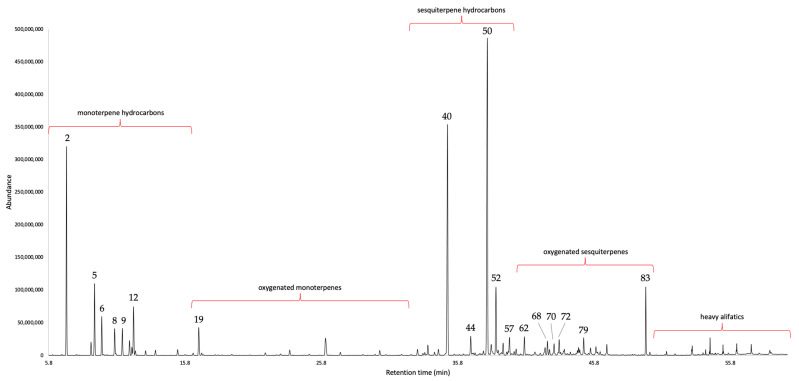
GC–MS profile of *G. laurifolia* EO on a 5%-phenyl-methylpolysiloxane stationary phase. The peak numbers refer to the compound numbers in Table 1. The approximate time ranges of each fraction are represented.

**Figure 2:**
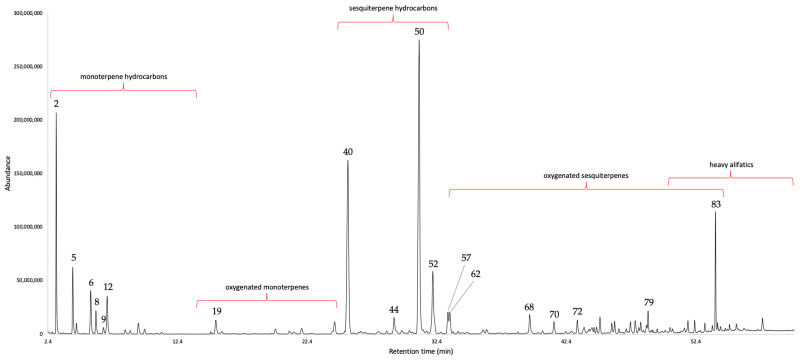
GC–MS profile of *G. laurifolia* EO on a polyethylene glycol stationary phase. The peak numbers refer to the compound numbers in Table 1. The approximate time ranges of each fraction are represented.

The steam-distillation of the leaves afforded a yellow oil that spontaneously separated from water. The yield was 0.02% by weight with respect to fresh plant material. After gas chromatographic (GC) analyses on two orthogonal columns, 90 constituents were detected and quantified with at least one column, corresponding to 95.9–95.0% by weight of the whole oil mass. The EO was dominated by sesquiterpene hydrocarbons (about 45%), followed by monoterpene hydrocarbons (about 25–30%). The major components (≥3.0%), on the non-polar and polar stationary phase, respectively, are: germacrene D (18.9–18.0%), (*E*)-β-caryophyllene (13.2–15.0%), α-pinene (11.0–10.3%), β-pinene (4.5–4.4%), β-phellandrene (4.0–3.0%), bicyclogermacrene (4.0–3.0%), and bakkenolide A (3.2–3.4%). The results of the chemical analysis are shown in Table 1, whereas the GC profiles on both columns are represented in Figure 1 and Figure 2.

### 2.2. Enantioselective Analysis

**Figure 3:**
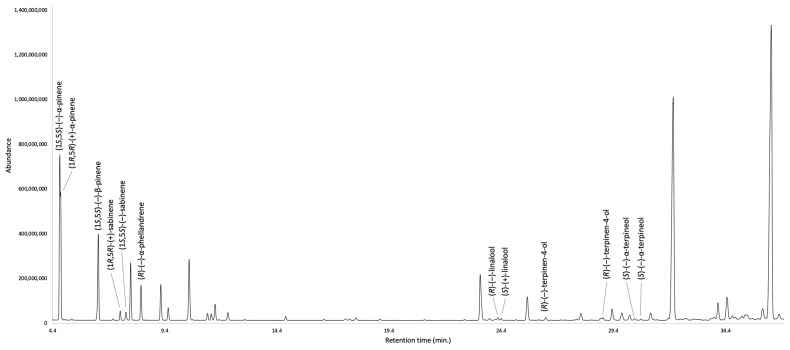
Enantioselective analysis of *G. laurifolia* EO on a 2,3-diacetyl-6-*tert*-butyldimethylsilyl-β-cyclodextrin stationary phase.

**Figure 4:**
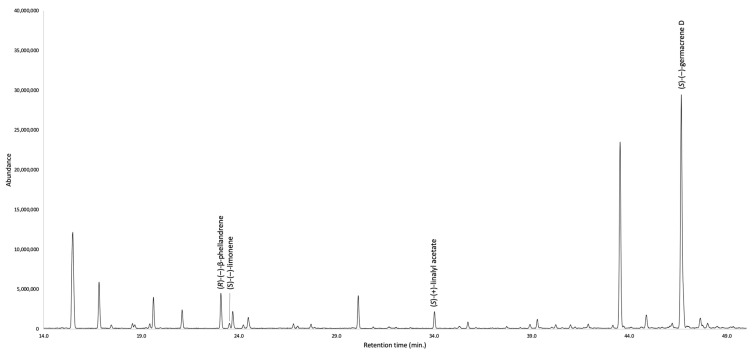
Enantioselective analysis of *G. laurifolia* EO on a 2,3-diethyl-6-*tert*-butyldimethylsilyl-β-cyclodextrin stationary phase.

The enantioselective analysis detected eleven chiral terpenes, whose enantiomers were separable on at least one of the two available enantioselective columns. In particular, the optical isomers of β-phellandrene, limonene, linalyl acetate, and germacrene D were better separated on a 2,3-diethyl-6-*tert*-butyldimethylsilyl-β-cyclodextrin stationary phase, whereas the enantiomers of all the other chiral components were easily resolved on a 2,3-diacetyl-6-*tert*-butyldimethylsilyl-β-cyclodextrin chiral selector. As a result, (1*S*,5*S*)-(−)-β-pinene, (*R*)-(−)-α-phellandrene, (*R*)-(−)-β-phellandrene, (*S*)-(−)-limonene, (*S*)-(+)-linalyl acetate, and (*S*)-(−)-germacrene D were identified as enantiomerically pure compounds, sabinene was detected as a racemic mixture, whereas all the other constituents are present as scalemic mixtures. As usual, the number of detected enantiomers is relatively small with respect to the total amount of chiral compounds present in the EO. This issue is due to the very limited commercial availability of chiral standards that, with few exceptions, are mainly constituted of enantiomerically pure monoterpenes. The detailed results are shown in Table 2, whereas the enantioselective GC profiles are represented in Figure 3 and Figure 4.

## 3. Discussion

The leaf EO of *G. laurifolia* was submitted for chemical analysis on two orthogonal columns. From the qualitative and quantitative point of view, the results were reciprocally consistent with both stationary phases, confirming the substantial correctness of these results. As mentioned in the introduction, the EOs of other *Gynoxys* spp. from the same region (*G. miniphylla*, *G. rugulosa*, and *G. buxifolia*) have been described in previous studies, as a part of the present project [16,17,18]. On the one hand, the volatile fraction of *G. buxifolia* was very different from all the others, including the one described in the present report. In fact, the distillation yield was quite high (0.1% *w*/*w*) and the main components were furanoeremofilane (about 30%) and bakkenolide A (about 17%), both very unusual EO constituents. On the other hand, *G. miniphylla* and *G. rugulosa* EOs were relatively similar. All these EOs presented an important sesquiterpene fraction, where germacrene D is always among the dominant compounds, followed by common sesquiterpenes, such as (*E*)-β-caryophyllene and δ-cadinene, among others. Furthermore, all these species produced a minority monoterpene fraction, where one or two hydrocarbons, usually α-pinene or α-phellandrene, were among the major components of the oil. Finally, *G. rugulosa* and *G. laurifolia* produced a heavy aliphatic fraction, absent in *G. buxifolia* and *G. miniphylla* but present in other *Gynoxys* EOs, whose analysis is currently in progress. According to the chemical composition, based on the major components, some biological properties of this volatile fraction could be predicted, but need to be confirmed in further investigations. With respect to this, the first most abundant compound was germacrene D. This very common sesquiterpene has not been exhaustively investigated so far for its biological activities as a pure substance, however it is well known for being an insect attractant for the tobacco budworm moth *Heliotis virescens* [21]. In particular, it has been demonstrated that this capacity, extended to *Helicoverpa armigera* and *Helicoverpa assulta*, is specific to the levorotatory isomer [22]. Interestingly, the enantioselective analysis conducted in the present study indicated that (*S*)-(−)-germacrene D is the only enantiomer present in *G. laurifolia* EO.

The second most abundant constituent is (*E*)-β-caryophyllene. In contrast to germacrene D, this extremely common sesquiterpene has been widely studied as a pure compound and its properties are described in the literature. In fact, (*E*)-β-caryophyllene has been described as an anti-inflammatory, neuroprotective, analgesic, antioxidant, sedative, anxiolytic, and antitumor compound. Some of these properties have been explained with the agonist action of (*E*)-β-caryophyllene on the CB2-R cannabinoid receptor, where this terpene interacts without exerting any psychotropic effect [23,24]. Nevertheless, according to the same literature, the anti-inflammatory and neuroprotective activities are probably the main properties of (*E*)-β-caryophyllene.

After that, α-pinene is the third most abundant metabolite and also a very common and deeply studied compound. This monoterpene also showed a very wide range of biological properties, such as antibacterial, antifungal, anti-leishmanial, anti-inflammatory, antioxidant, neuroprotective, antitumor, insecticidal, nematocidal, among others [25]. The most interesting activity is the anti-inflammatory capacity, since it basically coincides with the one for (*E*)-β-caryophyllene, with which it shares a similar mechanism (inhibition of NF-κB, TNF-α, and IL-6 mediators; suppression of MAPKs and NF-κB in mouse peritoneal macrophages; inhibition of iNOS and COX-2) [24,25]. As usual, the two optical isomers show different biological properties, the levorotatory enantiomer being anti-viral and the dextrorotatory neuroprotective due to its cholinergic activity [25]. According to our enantioselective analysis, *G. laurifolia* EO contained both enantiomers, with a 29.6% enantiomeric excess in favor of the anti-viral (1*S*,5*S*)-(−)-α-pinene.

The next main components are β-pinene, β-phellandrene, and bicyclogermacrene, each one accounting for about 4% of the whole oil mass. Despite that all these terpenes are almost ubiquitous in EOs, only β-pinene has been deeply investigated and described regarding its biological activities. In this respect, β-pinene presents many of the properties known for its constitutional isomer α-pinene, with the antibacterial activity appearing as the most important [26]. On the other hand, β-phellandrene and bicyclogermacrene have been mainly indirectly investigated, since most of the literature about these compounds deals with the biological activities of EOs rich in these compounds, instead of the pure terpenes. Nevertheless, if no clear conclusions can be found about β-phellandrene, an interesting property has been reported for bicyclogermacrene. In this case, purifying germacrene D and bicyclogermacrene from the EO of *Porcelia macrocarpa*, it has been demonstrated that the mixture of both sesquiterpenes exerted a cytotoxic activity against B16F10-Nex2, HCT, and HL-60 cancer cell lines greater than the sum of the single compounds, suggesting a non-linear synergic effect [27].

Finally, bakkenolide A must be mentioned. Despite being the less abundant of the major components (about 3%), this oxygenated sesquiterpene is absolutely the most interesting one, at least from the phytochemical point of view. In fact, whereas α-pinene, germacrene D, and (*E*)-β-caryophyllene are also the main components in almost all the EOs currently under investigation in the genus *Gynoxys*, bakkenolide A was only found so far in *G. buxifolia* [18]. On that occasion, the authors reported that the two main compounds (furanoeremofilane and bakkenolide A), despite being rare in EOs, are known to be often detected together in plants, due to biosynthetic reasons. However, in *G. laurifolia* EO, only bakkenolide A was present, whereas furanoeremofilane was not detected, even by selective ion-extraction in GC–MS. Furthermore, bakkenolide A is well known in the literature for three biological activities: a selective cytotoxic capacity against cancer cells, a promising anti-leukemic activity, and the antifeedant property versus *Sitophilus granarius*, *Tribolium confusum*, *Trogoderma granarium*, and *Peridroma saucia* [28,29,30,31].

The enantioselective analyses of the present investigation confirmed the usual existence of different biosynthetic pathways, devoted to the obtention of different enantiomers, in *G. laurifolia*. In fact, it is well known that the optical isomers of a chiral compound, despite presenting the same physicochemical properties, are characterized by different biological activities. In many volatile fractions, a typical case is the different odor of two optical isomers, which explains why two EOs of similar chemical composition can present a totally different aroma [32]. The different biological properties of the enantiomers, detected for the main components of *G. laurifolia* EO, have already been discussed in the present section. Furthermore, according to the authors’ experience, the number of enantiomerically pure chiral terpenes detected in this EO is a little unusually high.

Finally, some consideration should be taken regarding the plant’s availability and its possible large-scale applications. It must be remarked that this species is currently only wild, and it is classified as “vulnerable” in the Red Book of the endemic plants of Ecuador [33]. These aspects, together with the relatively low distillation yield, make an industrial application of this EO quite improbable, at least until *G. laurifolia* is made cultivable. Nevertheless, it has been previously clearly stated that the aim of the present study is not applicative. In the first instance, the focus of this investigation is the phytochemical description of the metabolic volatile fraction of *G. laurifolia* for an academic purpose.

## 4. Materials and Methods

### 4.1. Plant Material

The leaves of *G. laurifolia* were harvested on December 7, 2020, from eight distinct tiny shrubs that were situated 2650 m above sea level. The plants were spread out over a 200 m radius around a central position with the coordinates 03°59′48″S and 79°15′39″W. The location of the collection was in the Ecuadorian province of Loja, on the slopes of Mount Villonaco. The approximate identical weight of leaves from each shrub was collected, in order to create a single mean sample that was equally representative of all the plants. The entire fresh plant material (8.2 kg) was steam distilled the same day of collection. Based on a botanical sample with voucher 2850456, preserved at the National Museum of Natural History, Smithsonian Institution, Washington, DC, one of the authors (N.C.) carried out the plant identification. A specimen with the code 14770 was added to the herbarium of the Universidad Técnica Particular de Loja (UTPL), with the MAATE registry number MAE-DNB-CM-2016-0048. The collection and investigation was performed with the Ministry of Environment, Water, and Ecological Transition of Ecuador’s consent.

### 4.2. Plant Distillation and Sample Preparation

A stainless-steel Clevenger-type equipment was used to preparatively steam distill the entire amount of collected fresh plant material (8.2 kg). The EO was finally isolated from the aqueous phase after a 4 h-long operation. The EO was then kept at −15 °C in the dark until usage. For each GC analysis, 1 mL of cyclohexane standard solution (containing 0.7 mg/mL of *n*-nonane as the internal standard) was used to dilute about 10 mg of the EO. In all of the studies in the present work, the samples were prepared in this manner and then directly injected (1 μL) into the GC. Both cyclohexane and *n*-nonane were purchased from Sigma-Aldrich in St. Louis, MO, USA.

### 4.3. Qualitative GC–MS Analysis

A Trace 1310 gas chromatograph (Thermo Fisher Scientific, Walthan, MA, USA) and a simple quadrupole mass spectrometry detector (model ISQ 7000, both from Thermo Fisher Scientific) were used to analyze the EO qualitatively. The electron ionization (EI) source for the mass spectrometer was set to 70 eV, and the SCAN mode (scan range 40–400 *m*/*z*) was selected. The transfer line and ion source were both set to 230 °C. A non-polar stationary phase, based on 5% phenyl-methylpolysiloxane (DB-5ms), and a polar stationary phase, based on polyethylene glycol (HP-INNOWax), were applied for compound identification. Both columns were acquired from Agilent Technology (Santa Clara, CA, USA) and were 30 m long with, a 0.25 mm internal diameter and 0.25 m film thickness. Helium was used as the carrier gas, with a flow rate of 1 mL/min and the injector was programmed to run in SPLIT mode at 230 °C. The following thermal program was used to conduct the elution: 50 °C for 5 min, then a gradient of 2 °C/min to 100 °C, a second gradient of 3 °C/min to 150 °C, and a third gradient of 5 °C/min to 200 °C. Finally, a fourth ramp of 15 °C/min was used to raise the temperature to 230 °C, where it remained for 15 min. By comparing each linear retention index, calculated in accordance with Van den Dool and Kratz, and the associated mass spectrum with published data [18,19,20,34], the components of the EO were determined. The homologous series C9–C25 alkanes, used to calculate linear retention indices, were purchased from Sigma-Aldrich.

### 4.4. Quantitative GC–FID Analysis

The same GC, columns, instrument configuration, and thermal program used for the qualitative analysis were also used for the quantitative one. However, in this instance, a flame ionization detector (FID) was employed, which was fed with a combination of hydrogen and air at flows of 30 mL/min and 300 mL/min, respectively, and set to a temperature of 250 °C. Through the use of an internal standard (*n*-nonane) and two six-point calibration curves, all of the detected EO components were quantified on both columns. The corresponding relative response factors (RRFs) were determined in accordance with the combustion enthalpies [35,36]. Isopropyl caproate, the calibration standard, was synthesized at one of the authors’ laboratories (G.G.) and refined to 98.8% (GC–FID purity). The calibration standard solutions were prepared in accordance with earlier literature descriptions, yielding a correlation coefficient of 0.998 in both columns [10].

### 4.5. Enantioselective Analyses

The same GC–MS instrument described for the qualitative analysis was used for the enantioselective investigation. It was configured with two enantioselective columns, based on the chiral selectors 2,3-diethyl-6-*tert*-butyldimethylsilyl-cyclodextrin and 2,3-diacetyl-6-*tert*-butyldimethylsilyl-cyclodextrin. Both columns were 25 m × 0.25 mm × 0.25 μm (film thickness) and they were both purchased from Mega S.r.l., Legnano, Italy. The enantiomers were identified on the basis of their mass spectra and linear retention indices, compared with data obtained from a set of enantiomerically pure standards, available at the Pharmaceutical Biology research group of the University of Turin, Italy.

## 5. Conclusions

The fresh leaves of *Gynoxys laurifolia* (Kunth) Cass. produce an EO, with a distillation yield of 0.02% by weight. Due to its chemical composition, this oil is suitable to be submitted for further investigation as an insect attractive, anti-inflammatory, anticancer, and antifeedant agent. The enantioselective analyses confirmed the existence in this taxon, as in all the *Gynoxys* species studied so far, of enantioselective biosynthetic pathways. However, of the eleven chiral metabolites analyzed, six were unusually enantiomerically pure.

## Figures and Tables

**Table 1:** Qualitative and quantitative analyses of *G. laurifolia* EO with non-polar and polar columns.

N.	Identification	5%-Phenyl-Methylpolysiloxane					Polyethylene Glycol				
		LRI ^a^	LRI ^b^	%	σ	Reference	LRI ^a^	LRI ^b^	%	σ	Reference
1	α-thujene	924	924	0.1	0.02	[19]	1027	1020	0.1	0.01	[20]
2	α-pinene	932	932	11.0	0.02	[19]	1020	1028	10.3	0.57	[20]
3	α-fenchene	949	945	0.1	0.02	[19]	1062	1060	trace	-	[20]
4	sabinene	973	969	0.8	0.02	[19]	1118	1121	0.5	0.29	[20]
5	β-pinene	979	974	4.5	0.02	[19]	1107	1105	4.4	0.14	[20]
6	myrcene	991	988	0.7	0.02	[19]	1160	1167	0.6	0.04	[20]
7	dehydro-1,8-cineole	994	988	0.1	0.02	[19]	1185	1192	0.1	0.01	[20]
8	α-phellandrene	1009	1002	1.6	0.02	[19]	1204	1205	1.9	0.34	[20]
9	α-terpinene	1019	1014	1.6	0.06	[19]	1174	1179	1.2	0.07	[20]
10	*p*-cymene	1028	1020	1.3	0.06	[19]	1263	1268	1.1	0.04	[20]
11	limonene	1031	1024	0.8	0.06	[19]	1195	1192	0.6	0.09	[20]
12	β-phellandrene	1033	1025	4.0	0.06	[19]	1204	1205	3.0	0.13	[20]
13	1,8-cineol	1035	1026	0.5	0.06	[19]	1214	1220	0.3	0.27	[20]
14	(*E*)-β-ocimene	1048	1044	0.5	0.02	[19]	1247	1256	0.3	0.02	[20]
15	γ-terpinene	1060	1054	0.4	0.02	[19]	1238	1238	0.5	0.17	[20]
16	terpinolene	1088	1086	0.5	0.02	[19]	1275	1278	0.5	0.16	[20]
17	*p*-cymenene	1097	1089	trace	-	[19]	1428	1425	0.1	0.02	[20]
18	linalool	1106	1095	0.3	0.01	[19]	1552	1556	0.2	0.08	[20]
19	*n*-nonanal	1112	1100	2.8	0.11	[19]	1390	1387	2.6	0.18	[20]
20	2-(1-*Z*)-propenyl-phenol	1138	1146	0.1	0.02	[19]	-	-	-	-	-
21	prenyl isovalerate	1149	1147	0.1	0.02	[19]	-	-	-	-	-
22	(*E*)-2-nonenal	1169	1157	trace	-	[19]	1632	1642	0.1	0.02	[20]
23	terpinen-4-ol	1187	1174	0.3	0.03	[19]	1592	1589	0.1	0.09	[20]
24	α-terpineol	1204	1186	0.1	0.04	[19]	1679	1675	0.1	0.04	[20]
25	*n*-decanal	1215	1201	0.6	0.04	[19]	1491	1501	0.4	0.3	[20]
26	thymol methyl ether	1237	1232	0.1	0.04	[19]	1588	1586	trace	-	[20]
27	linalyl acetate	1255	1254	1.1	0.04	[19]	1564	1569	1.0	0.07	[20]
28	carvona	1257	1239	1.3	0.04	[19]	1699	1704	0.8	0.28	[20]
29	(*E*)-2-decenal	1273	1260	0.4	0.04	[19]	1632	1630	0.6	0.02	[20]
30	*n*-undecanal	1316	1305	0.1	0.04	[19]	1597	1598	0.1	0.01	[20]
31	*p*-vinylguaiacol	1323	1309	0.3	0.04	[19]	2193	2197	0.6	0.07	[20]
32	δ-elemene	1332	1335	trace	-	[19]	1453	1452	0.1	0.01	[20]
33	α-terpineol acetate	1353	1346	0.1	0.01	[19]	1651	1650	0.1	0.03	[20]
34	geranyl acetate	1366	1379	0.2	0.01	[19]	1719	1717	0.2	0.1	[20]
35	α-ylangene	1376	1373	0.5	0.01	[19]	1474	1472	1.2	0.04	[20]
36	bourbonene	1383	1387	0.1	0.01	[19]	1497	1496	trace	-	[20]
37	β-elemene	1390	1389	1.5	0.16	[19]	1576	1575	1.0	0.31	[20]
38	cyperene	1406	1400	0.6	0.16	[19]	1519	1520	0.3	0.24	[20]
39	α-cedrene	1417	1410	trace	-	[19]	1566	1566	0.1	0.01	[20]
40	(*E*)-β-caryophyllene	1420	1417	13.2	1.42	[19]	1574	1575	15.0	0.47	[20]
41	β-copaene	1431	1430	0.1	1.42	[19]	1566	1565	0.3	0.02	[20]
42	aromadendrene	1440	1439	0.1	1.42	[19]	1618	1622	0.3	0.06	[20]
43	spirolepechinene	1446	1449	0.3	1.42	[19]	1644	-	0.7	0.55	§
44	α-humulene	1457	1452	1.5	1.42	[19]	1643	1644	1.6	0.05	[20]
45	alloaromadendrene	1462	1458	trace	-	[19]	1617	1618	0.1	0.01	[20]
46	*cis*-cadina-1(6),4-diene	1465	1461	0.5	0.02	[19]	1768	1778	0.1	0.09	[20]
47	1,5-di-*epi*-aristolochene	1472	1471	0.3	0.02	[19]	1657	-	0.4	0.14	§
48	β-chamigrene	1475	1476	0.2	0.02	[19]	1697	1686	0.1	0.02	[20]
49	γ-muurolene	1478	1478	0.6	0.02	[19]	1665	1668	0.2	0.2	[20]
50	germacrene D	1484	1480	18.9	0.02	[19]	1683	1684	18.0	0.57	[20]
51	γ-amorphene	1490	1495	0.7	0.02	[19]	1695	1693	0.4	0.23	[20]
52	bicyclogermacrene	1498	1500	4.0	0.02	[19]	1714	1706	3.0	0.1	[20]
53	α-muurolene	1501	1500	trace	-	[19]	1720	1723	0.1	0.08	[20]
54	α-bulnesene	1512	1509	0.4	0.05	[19]	1615	1618	0.8	0.23	[20]
55	γ-cadinene	1517	1513	trace	-	[19]	1712	1716	0.9	0.03	[20]
56	*n*-tridecanal	1519	1509	0.2	0.01	[19]	1807	1809	0.3	0.17	[20]
57	δ-cadinene	1522	1522	1.0	0.01	[19]	1738	1744	0.3	0.04	[20]
58	*cis*-calamenene	1526	1528	0.1	0.01	[19]	1809	1814	trace	-	[20]
59	(*E*)-γ-macrocarpene	1530	1527	0.2	0.01	[19]	1815	-	0.1	0.02	§
60	kessane	1534	1529	0.4	0.01	[19]	1830	-	0.1	0.04	§
61	α-cadinene	1542	1537	0.1	0.01	[19]	1767	1769	trace	-	[20]
62	undetermined (MW 220)	1549	-	1.2	0.05	-	1741	-	0.6	0.22	-
63	*cis*-cadinene ether	1552	1552	0.1	0.01	[19]	2010	-	0.1	0.02	§
64	(*E*)-nerolidol	1567	1561	0.3	0.01	[19]	2042	2053	0.2	0.04	[20]
65	gleenol	1577	1586	0.1	0.01	[19]	2035	2032	0.1	0.01	[20]
66	germacrene-4-ol	1583	1574	0.2	0.01	[19]	2035	2050	0.3	0.01	[20]
67	spathulenol	1585	1577	0.7	0.01	[19]	2108	2106	0.3	0.25	[20]
68	caryophyllene oxide	1590	1582	1.1	0.01	[19]	1933	1940	1.3	0.05	[20]
69	globulol	1593	1590	0.4	0.01	[19]	1999	2010	0.3	0.04	[20]
70	viridiflorol	1602	1592	0.9	0.03	[19]	2065	2062	1.1	0.04	[20]
71	cubeban-11-ol	1604	1594	0.4	0.03	[19]	2018	-	0.8	0.18	§
72	undetermined (MW 222)	1613	-	1.0	0.05	-	2058	-	0.9	0.08	-
73	tetradecanal	1623	1611	0.2	0.03	[19]	1921	1921	0.3	0.01	[20]
74	di-*epi*-1,10-cubenol	1624	1618	0.4	0.03	[19]	2053	2054	0.6	0.04	[20]
75	alloaromadendrene epoxide	1646	1639	trace	-	[19]	1650	1646	0.7	0.03	[20]
76	α-*epi*-cadinol	1654	1638	0.1	0.03	[19]	2156	2170	0.7	0.03	[20]
77	α-cadinol	1656	1652	0.1	0.03	[19]	2215	2218	0.7	0.03	[20]
78	α-muurolol	1659	1644	0.1	0.03	[19]	2161	2165	0.5	0.1	[20]
79	undetermined (MW 222)	1668	-	1.1	0.16	-	2217	-	1.0	0.03	-
80	khushinol	1683	1679	0.8	0.03	[19]	2227	-	0.5	0.03	§
81	α-bisabolol	1699	1685	trace	-	[19]	2212	2214	0.5	0.05	[20]
82	shyobunol	1705	1688	0.1	0.04	[19]	1935	1930	0.5	0.04	[20]
83	bakkenolide A	1851	1845	3.2	0.04	[18]	2428	2430	3.4	0.12	[18]
84	*n*-nonadecane	1900	1900	0.3	0.05	[19]	1900	1900	0.4	0.01	[20]
85	1-eicosene	1995	1987	0.1	0.02	[19]	1980	-	0.1	0.04	§
86	*n*-heneicosane	2100	2100	0.5	0.04	[19]	2100	2100	0.5	0.02	[20]
87	1-docosene	2193	2189	0.1	0.01	[19]	2205	-	0.1	0.02	§
88	*n*-tricosane	2300	2300	0.3	0.01	[19]	2300	2300	0.1	0.01	[20]
89	*n*-tetracosane	2400	2400	0.1	0.01	[19]	2400	2400	0.5	0.01	[20]
90	*n*-pentacosane	2500	2500	0.1	0.01	[19]	2500	2500	trace	-	[20]
	monoterpene hydrocarbons			27.9					25.1		
	oxygenated monoterpenes			4.2					2.9		
	sesquiterpene hydrocarbons			45.3					45.2		
	oxygenated sesquiterpenes			12.3					15.1		
	others			6.2					6.7		
	total			95.9					95.0		

^a^ Calculated linear retention index; ^b^ Reference linear retention index; % = percentage by weight; σ = standard deviation; § = identified by mass spectrum only.

**Table 2:** Enantioselective analysis of *G. laurifolia* EO on two β-cyclodextrin-based chiral selectors.

Enantiomers	LRI	Enantiomeric Distribution (%)	*e.e.* (%)
(1*S*,5*S*)-(−)-α-pinene	925 *	64.8	29.6
(1*R*,5*R*)-(+)-α-pinene	926 *	35.2	
(1*S*,5*S*)-(−)-β-pinene	979 *	100.0	100.0
(1*R*,5*R*)-(+)-sabinene	1006 *	49.8	0.4
(1*S*,5*S*)-(−)-sabinene	1012 *	50.2	
(*R*)-(−)-α-phellandrene	1026 *	100.0	100.0
(*R*)-(−)-β-phellandrene	1050 **	100.0	100.0
(S)-(−)-limonene	1057 **	100.0	100.0
(*S*)-(+)-linalyl acetate	1257 **	100.0	100.0
(*R*)-(−)-linalool	1305 *	62.3	24.6
(*S*)-(+)-linalool	1307 *	37.7	
(*R*)-(−)-terpinen-4-ol	1339 *	53.0	6.0
(*S*)-(+)-terpinen-4-ol	1379 *	47.0	
(*S*)-(−)-α-terpineol	1402 *	66.3	32.6
(*R*)-(+)-α-terpineol	1407 *	33.7	
(*S*)-(−)-germacrene D	1467 **	100	100

LRI = linear retention index; *e.e.* = enantiomeric excess; * 2,3-diacetyl-6-*tert*-butyldimethylsilyl-β-cyclodextrin; ** 2,3-diethyl-6-*tert*-butyldimethylsilyl-β-cyclodextrin.

## Data Availability

Raw data are available from the authors (L.R.L.).
